# The transcriptional cascade CiERF023*-*CiERF041 of *Citrus ichangensis* regulates small heat shock protein gene *CiHSP26.5* to modulate cold tolerance

**DOI:** 10.1093/hr/uhag131

**Published:** 2026-04-07

**Authors:** Xi Zeng, Lele Chu, Wenqiang Hu, Yilei Wang, Yike Zeng, Peng Xiao, Jing Qu, Bachar Dahro, Wei Li, Shiping Zhu, Chunlong Li, Ji-Hong Liu

**Affiliations:** National Key Laboratory for Germplasm Innovation and Utilization of Horticultural Crops, College of Horticulture and Forestry Sciences, Huazhong Agricultural University, Wuhan 430070, China; National Key Laboratory for Germplasm Innovation and Utilization of Horticultural Crops, College of Horticulture and Forestry Sciences, Huazhong Agricultural University, Wuhan 430070, China; National Key Laboratory for Germplasm Innovation and Utilization of Horticultural Crops, College of Horticulture and Forestry Sciences, Huazhong Agricultural University, Wuhan 430070, China; National Key Laboratory for Germplasm Innovation and Utilization of Horticultural Crops, College of Horticulture and Forestry Sciences, Huazhong Agricultural University, Wuhan 430070, China; National Key Laboratory for Germplasm Innovation and Utilization of Horticultural Crops, College of Horticulture and Forestry Sciences, Huazhong Agricultural University, Wuhan 430070, China; National Key Laboratory for Germplasm Innovation and Utilization of Horticultural Crops, College of Horticulture and Forestry Sciences, Huazhong Agricultural University, Wuhan 430070, China; National Key Laboratory for Germplasm Innovation and Utilization of Horticultural Crops, College of Horticulture and Forestry Sciences, Huazhong Agricultural University, Wuhan 430070, China; National Key Laboratory for Germplasm Innovation and Utilization of Horticultural Crops, College of Horticulture and Forestry Sciences, Huazhong Agricultural University, Wuhan 430070, China; National Key Laboratory for Germplasm Innovation and Utilization of Horticultural Crops, College of Horticulture and Forestry Sciences, Huazhong Agricultural University, Wuhan 430070, China; Citrus Research Institute, Southwest University, Chinese Academy of Agricultural Sciences, Chongqing 400712, China; National Key Laboratory for Germplasm Innovation and Utilization of Horticultural Crops, College of Horticulture and Forestry Sciences, Huazhong Agricultural University, Wuhan 430070, China; Hubei Hongshan Laboratory, Wuhan 430070, China; National Key Laboratory for Germplasm Innovation and Utilization of Horticultural Crops, College of Horticulture and Forestry Sciences, Huazhong Agricultural University, Wuhan 430070, China; Hubei Hongshan Laboratory, Wuhan 430070, China

## Abstract

Ichang papeda (*Citrus ichangensis*), a wild primitive citrus species, exhibits remarkable cold tolerance, yet the transcriptional regulation of cold-responsive genes in *C. ichangensis* remains poorly explored. In this study, we identified the small heat shock protein (sHSP) *CiHSP26.5* as a crucial member that was substantially induced under cold stress and played a positive role in cold tolerance. Furthermore, two APETALA2/Ethylene Responsive Factor (AP2/ERF) family transcription factors, CiERF023 and CiERF041, were found to directly bind to the dehydration-responsive element (DRE) motifs in the promoter of *CiHSP26.5* and function as its negative and positive regulators, respectively. In addition, CiERF023 was proven to repress *CiERF041* through interacting with the DRE element in the promoter. These two TFs were differentially induced during cold stress, with *CiERF023* peaking at early stages and *CiERF041* at later stages. Consistently, *CiERF023* acts as a negative regulator, whereas *CiERF041* serves as a positive regulator, of cold tolerance by regulating *CiHSP26.5*. Taken together, our findings reveal that CiERF023 and CiERF041 form a hierarchical transcriptional cascade that antagonistically regulates *CiHSP26.5* expression under cold stress in *C. ichangensis*. This study provides fresh insights into the regulatory network governing HSP-mediated cold adaptation and identifies potential targets for enhancing cold tolerance in plants.

## Introduction

As sessile organisms, plants are constantly challenged by various environmental stresses, including drought, salinity, and extreme temperatures that are detrimental to their growth and development, yield, and quality [1–3]. Cold stress, one of the most serious abiotic stresses, primarily damages plant tissues, disrupting their physiological functions and ultimately leading to plant death. This is mainly manifested in root damage [4], accumulation of reactive oxygen species (ROS) within plants [5], inhibition of photosynthetic efficiency [6], and destruction of leaf cell structures [7], among others.

Through a long evolutionary process, plants have developed a complex mechanism to resist cold stress and maintain normal growth and development. This includes sensing cold signals [8], initiating signal transduction [9, 10], adaptive changes in the cell membrane system [11, 12], adjusting gene expression in signaling pathways [13], synthesizing osmotic regulators [14, 15], and enhancing the expression of molecular chaperones [16]. It has been documented that both biotic and abiotic stresses may promote the accumulation of unfolded, misfolded, or denatured proteins in cells, leading to the disruption of protein homeostasis and impairment of plant survival [17–19]. In order to combat this disorder, plants have evolved a highly conserved and efficient defense system, in which molecular chaperones play a crucial role. As one of the major categories of molecular chaperones, heat shock proteins (HSPs) are responsible for protein folding, assembly, translocation, and degradation during cellular processes and thus play a crucial role in stabilizing proteins and membranes under adverse conditions [20]. Under abiotic stresses, multiple members of the HSP family exert pivotal functions in enhancing stress tolerance and sustaining cell survival across different species. For example, the tomato chloroplast HSP21 acted as a molecular chaperone to protect PSII under oxidative stresses [21]. The upregulation of *HSP101* in *Arabidopsis* has been shown to be necessary for its acquisition of heat tolerance [22]. sHSP17.4 of maize was demonstrated to play a positive role in modulation of heat tolerance [23]. In addition, a plasma membrane-localized Hsp40-encoding gene *OsDNAJ15* of rice translocated to the nucleus, where it could interact with OsBAG4 to enhance OsMYB106 DNA-binding activity under salt stress [24]. While these findings underpin the understanding of the vital function of HSPs in plant abiotic stress tolerance, there is a great paucity of knowledge on the regulatory network of molecular chaperones under cold stress. Of note, few molecular regulators that function to control the molecular chaperones have been deciphered. Therefore, further investigations are indispensable to fully clarify the transcriptional regulatory mechanisms that govern their abiotic stress-induced responses.

In response to environmental stress, plants mobilize a complex network of transcriptional regulators, including transcription factors (TFs), protein kinases, and protein phosphatases, to mitigate stress-induced damages. Gene regulation is a dynamic process in which the TFs play a crucial role in regulating the spatiotemporal expression of the stress-responsive genes [25]. APETALA2/Ethylene Responsive Factors (AP2/ERFs) are critical regulators in responses to abiotic stresses by targeting downstream genes through binding to the motifs, including the GCC box or DRE (dehydration-responsive element)/CRT (C-repeat), within the promoter regions [26]. Accumulating evidence demonstrates that different members of AP2/ERF TFs exhibit functional specificity and diversity in stress responses. PtrERF108 has been demonstrated to directly regulate the expression of *PtrRafS* to modulate RafS activity and raffinose content for enhancing cold tolerance [27]. *LbAP2/ERF32* of *Limonium bicolor* L. positively regulated salt tolerance by promoting salt gland development and salt secretion [28]. ZmDREB2A of maize was reported to mediate gene expression responses to both drought and heat stress [29]. LlERF110 of *Lilium longiflorum* could activate heat stress response-associated repressors and indirectly impair the normal expression of heat stress protection genes like *AtHSFA2*, thereby mitigating the heat tolerance [30]. Although these ERFs act as positive regulators or transcriptional activators, ERFs have been also shown to serve as transcriptional repressors of a myriad of genes in different processes. For instance, ERF.D2 of tomato could bind the promoters of key jasmonic acid (JA) biosynthesis genes *AOC* and *OPR3* for repressing their transcription to reduce endogenous JA levels and maintain plant growth [31]. MdERF4 of apple negatively regulated the salt stress response by directly inhibiting the expression of *MdERF3* [32]. Soybean GmERFA repressed the expression of *GmZF392* and *GmbZIP123*, consequently impeding oil biosynthesis [33]. Despite the numerous studies concerning the functional regulation of ERFs on a large spectrum of target genes, it remains elusive whether and how ERFs regulate HSPs in response to cold stress. In addition, antagonistic regulation of a given target by both ERFs acting as activators and repressors is far from being well illustrated.

Citrus is the world’s largest fruit and also the most widely cultivated and economically significant fruit tree in southern China. As a tropical fruit tree, citrus is highly susceptible to cold stress. Low temperature stress can reduce the photosynthetic parameters and promote lipid peroxidation of citrus leaves [34]. In more severe cases, it can lead to the formation of ice crystals in the intercellular spaces of tissues, accompanied by the rupture of cell membranes and damage to cell walls, causing significant injury to citrus vascular tissues, leaves, tree crowns, and fruits [35, 36]. This severely inhibits the growth and development of citrus, reduces fruit yield, and restricts industrial development. Substantial variation in cold tolerance exists among different citrus accessions. For instance, trifoliate orange (*Poncirus trifoliata*) and wild citrus generally exhibit considerably greater cold tolerance than cold-sensitive cultivars like lemon and pummelo [37, 38]. In recent years, the increasing frequency of extreme weather events has resulted in substantial losses to the citrus industry. Consequently, enhancing cold tolerance in citrus, through both conventional and modern biotechnological approaches, has become critically important. Ichang papeda is a primitive wild citrus species with excellent cold resistance. Although the cold stress adaptation mechanisms of Ichang papeda have been increasingly elucidated at the epigenetic and metabolic levels [37, 38], functional characterization and regulatory modules of the crucial genes closely involved in cold tolerance remain largely limited. Based on the previous transcriptome of cold-treated Ichang papeda [37], *CiHSP26.5* was found to be substantially induced by low temperature. In this study, we elucidated the positive function of *CiHSP26.5* in cold tolerance through the combined use of overexpression and virus-induced gene silencing (VIGS). Subsequently, two ERFs, CiERF023 and CiERF041, were confirmed to act as a transcriptional repressor and an activator of *CiHSP26.5*, respectively, by binding to the DRE motifs in the promoter. In addition, CiERF023 was shown to regulate the expression of *CiERF041*. Consistently, CiERF023 and CiERF041 functioned negatively and positively, respectively, to modulate cold tolerance. Interestingly, *CiERF023* exhibited a different expression pattern relative to that of *CiERF041* and *CiHSP26.5*, implying that the two ERFs might regulate *CiHSP26.5* to coordinate the cold stress responses in an antagonistic manner. Taken together, our work unveils the molecular module composed of CiERF023–CiERF041–*CiHSP26.5* to orchestrate the cold stress response and tolerance in Ichang papeda. These findings provide important insight into the elucidation of transcriptional regulation of HSPs under cold stress and provide valuable genes that hold great potential for enhancing cold tolerance through genetic engineering.

## Results

### Low temperature induces small heat shock protein expression

**Figure 1:**
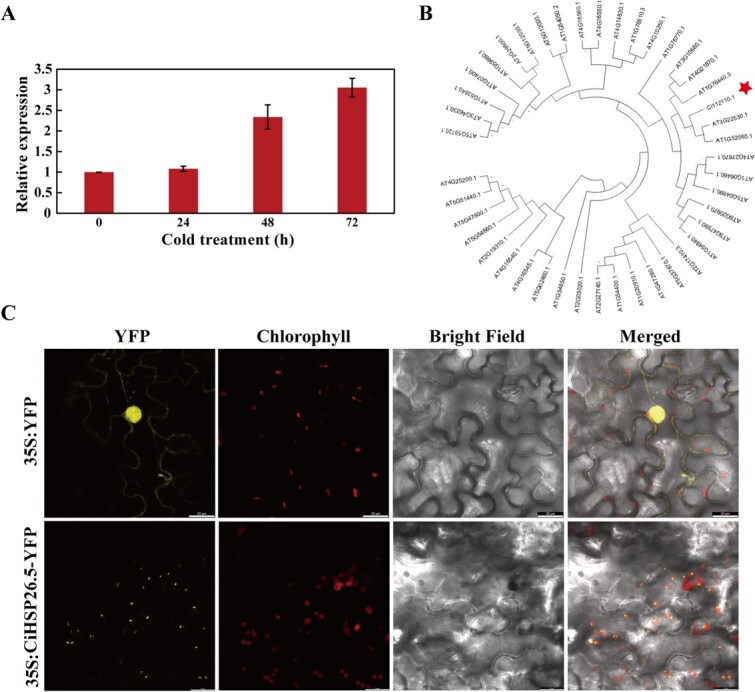
Low-temperature expression pattern, phylogenetic tree, and subcellular localization of CiHSP26.5. (A) Transcriptional levels of *CiHSP26.5* at different time points under 4°C cold stress. Actin served as an internal control, error bars indicate ± SD (*n* = 3). (B) Phylogenetic tree of *CiHSP26.5* and *A. thaliana HSP20* genes. (C) Subcellular localization analysis of CiHSP26.5. Confocal images were acquired in bright field mode or at the YFP/chloroplast fluorescence wavelength, with the overlay displayed on the right.

In our earlier RNA-seq data for Ichang papeda [37], *Ci112110* annotated as an *HSP* gene, was significantly induced by cold stress. Consistent with the RNA-seq, real-time quantitative polymerase chain reaction (RT-qPCR) showed that the mRNA abundance of *Ci112110* gradually and steadily increased and reached its peak level at 72 h after cold exposure (Fig. 1A). By constructing a phylogenetic tree using *Ci112110* and the *HSP* genes of *Arabidopsi*s *thaliana*, *Ci112110* was grouped together with *At1g52560.1 and At3g22530.1* in an independent branch, showing greater similarity to the former. Therefore, *Ci112110* was named *CiHSP26.5* hereafter (Fig. 1B). To investigate the subcellular location where CiHSP26.5 exerts its function, we generated a construct 35S:CiHSP26.5-YFP and transiently expressed it in *Nicotiana benthamiana* leaves. Compared with the ubiquitous distribution of 35S:YFP in the cell, the yellow fluorescent protein (YFP) signal of 35S:CiHSP26.5-YFP was exclusively detected in the chloroplasts, which was corroborated by the colocalization with chloroplast autofluorescence (Fig. 1C), implying *CiHSP26.5* is a chloroplast-localized small heat shock protein (sHSP).

### *CiHSP26.5* plays a positive role in cold tolerance

**Figure 2:**
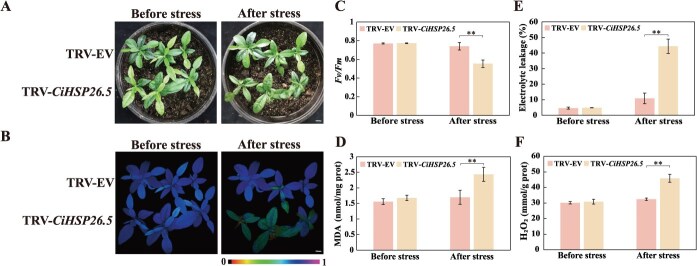
Knockdown of the *CiHSP26.5* in Ichang papeda via VIGS reduces cold tolerance. (A and B) Phenotypic and chlorophyll fluorescence images of the TRV-*CiHSP26.5* and TRV-EV control before and after low-temperature treatment, (C) *Fv/Fm* ratio, (D) MDA content, (E) EL, and (F) H_2_O_2_ content. Data are presented as mean ± SE (*n* = 3). Scale bars represent 1 cm for the images in the same panel.

The upregulation of *CiHSP26.5* in response to cold stress suggests that it may play a key role in cold tolerance. To confirm this assumption, *CiHSP26.5* was silenced in Ichang papeda by VIGS. The expression levels of *CiHSP26.5* were decreased in the VIGS plant compared to the control transformed with the tobacco rattle virus empty vector (TRV-EV) (Fig. S1). Under normal conditions, the TRV-EV and VIGS plants were similar in plant growth status. However, after the cold treatment and following recovery, the VIGS plants exhibited significant leaf withering, particularly the young leaves on the top (Fig. 2A). Chlorophyll fluorescence of the VIGS plants was substantially attenuated, concurrent with the significant decrease of *Fv/Fm* ratio, relative to the TRV-EV plants (Fig. 2B and C). Despite no differences in electrolyte leakage (EL), malondialdehyde (MDA), and hydrogen peroxide (H_2_O_2_) under normal conditions between the tested plants, these parameters were significantly higher in the VIGS plants when compared to the TRV-EV control in the presence of cold treatments (Fig. 2D–F).

**Figure 3:**
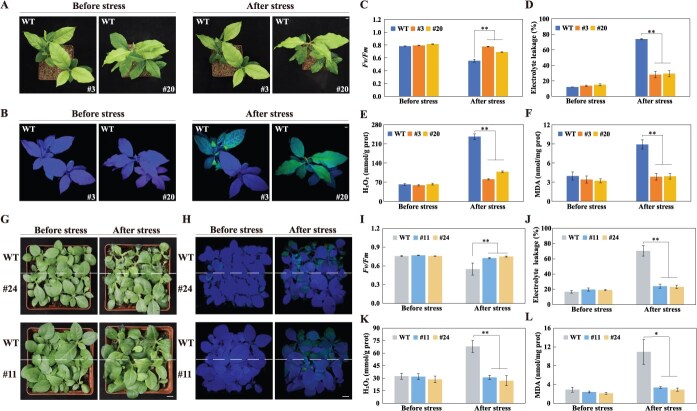
Overexpression of *CiHSP26.5* enhances the cold tolerance in transgenic lemons and tobacco. (A–F) Evaluation of cold tolerance in overexpressing lemons (#3 and #20), (A and B) phenotypic and chlorophyll fluorescence images of the transgenic lemons and WT before and after low-temperature treatment, (C) *Fv/Fm* ratio, (D) EL, (E) H_2_O_2_ content, and (F) MDA content. (G–L) Evaluation of cold tolerance in overexpressing tobacco (#24 and #11), (G and H) phenotypic and chlorophyll fluorescence images of the transgenic tobacco and WT control before and after low-temperature treatment, (I) *Fv/Fm* ratio, (J) EL, (K) H_2_O_2_ content, and (L) MDA content. Data are presented as mean ± SE (*n* = 3). Scale bars represent 1 cm for the images in the same panel.

To further assess the function of *CiHSP26.5*, two independent *CiHSP26.5*-overexpressing (OE) transgenic lemon (*Citrus limon*) lines (#3 and #20) were generated using *Agrobacterium*-mediated genetic transformation of lemon shoot segments (Fig. S2A). Under normal growth conditions, no significant phenotypic differences were observed between wild type (WT) and the two OE plants. However, when subjected to cold treatment and subsequent recovery, serious water soaking and leaf wilting were observed in the WT, while the transgenic lines suffered from less severe symptoms (Fig. 3A). The better growth phenotype of the transgenic lines was supported by stronger chlorophyll fluorescence and higher *Fv/Fm* ratio in comparison with the WT (Fig. 3B and C). In addition, significantly lower contents of EL, H_2_O_2_, and MDA were detected in the transgenic plants relative to the WT counterpart (Fig. 3D–F). Moreover, *CiHSP26.5*-OE transgenic tobacco lines were also produced to investigate the effects of ectopic overexpression on cold tolerance (Fig. S2B). The transgenic and WT tobacco plants were indistinguishable from each other in terms of plant morphology under normal conditions. When exposed to cold stress conditions, the transgenic tobacco plants exhibited prominently better growth status than those of the WT (Fig. 3G), which was further supported by observing the stronger chlorophyll fluorescence and higher *Fv/Fm* ratio (Fig. 3H and I), and reduction of EL, H_2_O_2_, and MDA content (Fig. 3J–L). Taken together, these results indicate that *CiHSP26.5* functions positively in modulation of cold tolerance.

### CiERF023 and CiERF041 transcriptionally regulate *CiHSP26.5*

**Figure 4:**
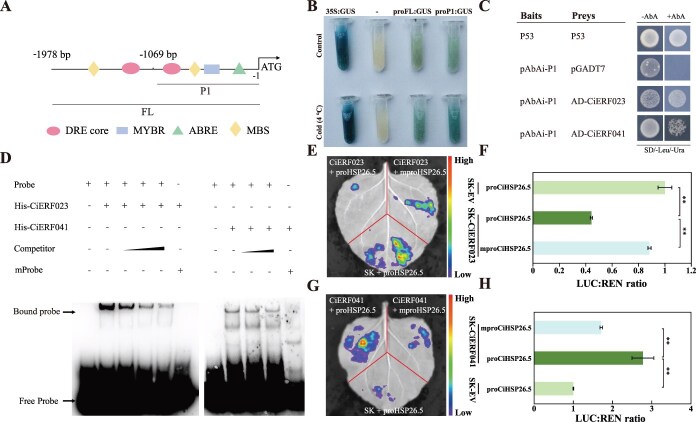
CiERF023 and CiERF041 bind to the promoter of *CiHSP26.5*, inhibiting and activating its transcriptional regulation, respectively. (A) Schematic diagram and distribution of cis-acting elements within the promoter of *CiHSP26.5*. FL represents the full-length promoter (−1978 bp), and P1 represents a partial fragment (−1069 bp) of the *CiHSP26.5* promoter. (B) GUS staining of calli transiently transformed with the *CiHSP26.5* Promoter. (C) Growth of the yeast cells co-transfected with prey (AD-CiERF023 or AD-CiERF041) and bait (pAbAi-P1), including negative control (pAbAi-P1 + pGADT7) and positive control (p53) on SD/-Ura/-Leu selective medium with 150 ng/ml AbA. (D) Detection of CiERF023 and CiERF041 binding to the DRE element (GCCGAC) of the P1 via EMSA. (E–H) LUC bioluminescence imaging (E and G) and dual LUC assays (F and H) were performed in tobacco leaves. Co-transfected with different effectors, including SK, SK-CiERF023 (E and F), SK-CiERF041 (G and H), and the promoter of *CiHSP26.5.* Data are presented as mean ± SD (*n* = 3).

To understand the molecular mechanism underlying the cold stress response of *CiHSP26.5*, we obtained a 1978-bp promoter sequence upstream of the transcription start site of *CiHSP26.5*. Bioinformatics analysis of the promoter sequence revealed the presence of several stress-related cis-acting elements, such as DRE, MYBR, ABRE, and MBS (Fig. 4A). In addition, the *GUS* gene driven by the full-length promoter and a truncated promoter fragment (P1) was transiently expressed in Satsuma mandarin calli to test the promoter activity. The results showed that GUS activity of the calli expressing both the full-length promoter and P1 fragment was significantly elevated by cold treatment, with the greatest activation being observed in the P1 fragment (Fig. 4B). These results suggest that the P1 sequence is important for the upregulation of *CiHSP26.5* under cold stress.

In order to unravel the transcriptional regulators of *CiHSP26.5*, the P1 fragment was employed to construct a yeast one-hybrid (Y1H) bait for screening a complementary DNA (cDNA) library. Five potential TFs were identified (Table S2), among which two were annotated as *CiERF023* and *CiERF041*. Efforts were then made to confirm the interaction of CiERF023 and CiERF041 with the promoter of *CiHSP26.5.* Y1H assay showed that all of the yeast cells grew well on SD/-Leu/-Ura medium. However, in the presence of Aureobasidin A (AbA), only the yeast cells of positive control and those co-transformed with the two preys (AD-CiERF023 or AD-CiERF041) and the P1 bait containing the putative DRE sequence could survive (Fig. 4C), implying that CiERF023 and CiERF041 interact with the promoter of *CiHSP26.5*. Electrophoretic mobility shift assay (EMSA) indicated that incubation of fusion protein of His-CiERF041 or His-CiERF023 and probes containing the DRE motif led to formation of protein–DNA complexes, whereas the migration was conspicuously impaired by the addition of the competitor DNA in a dosage-dependent manner. In addition, the band shift was completely abolished when the DRE sequence was mutated from GCCGAC to AAAAAA (Fig. 4D). The dual luciferase (LUC) assay showed that the promoter activity of *CiHSP26.5*, as indicated by the LUC:REN ratio, was elevated by co-expression of CiERF041 effector, but decreased by CiERF023. Of note, mutation of the DRE sequences in the promoter sequence led to resumption of the activity close to the control level (Fig. 4F and H). The quantitative measurements of LUC/REN ratio were supported by LUC fluorescence imaging of tobacco leaves infiltrated with the corresponding constructs (Fig. 4E and G). These results indicate that CiERF023 and CiERF041 act as a transcriptional repressor and an activator, respectively, of *CiHSP26.5* by directly binding to the promoter.

### CiERF023 and CiERF041 function as cold and ethylene-responsive transcription factors

**Figure 5:**
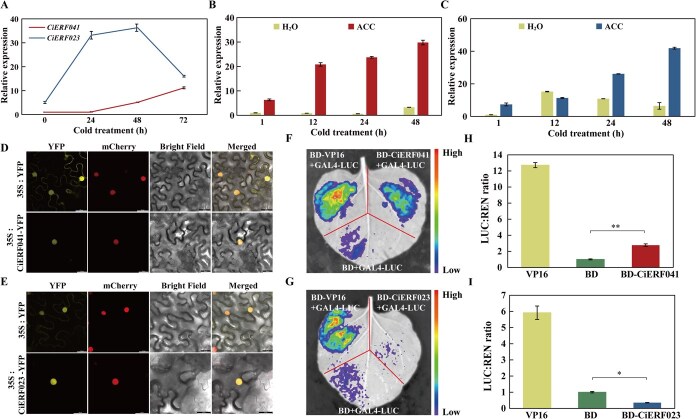
Expression, subcellular localization, and transcriptional activation analyses of *CiERF023* and *CiERF041*. (A) The expression patterns of *CiERF023* and *CiERF041* in response to low temperature. (B and C) The expression levels of *CiERF041* (B) and *CiERF023* (C) under H_2_O and ACC treatment. (D and E) Subcellular localization analysis of CiERF041 (D) and CiERF023 (E). Confocal images were acquired in bright field mode or at the YFP/mCherry fluorescence wavelength, with the overlay displayed on the right. (F–I) Dual LUC assays (H and I) and LUC bioluminescence imaging (F and G) were performed in tobacco leaves. Co-transfected with different effectors, including BD-VP16, BD-CiERF041 (F and H), BD-CiERF023 (G and I), and the reporter of TATA*.* Data are presented as mean ± SD (*n* = 3).

To further elucidate the functions of *CiERF023* and *CiERF041,* we analyzed their expression patterns under low temperature stress. The transcript level of *CiERF023* was quickly induced at 24 h of cold treatment, reaching the peak (7.2-fold upregulation) at 48 h. By contrast, the mRNA abundance of *CiERF041* remained stable within 24 h of cold treatment, and then increased at 48 and 72 h, when the expression level was increased by nearly 10 folds relative to the initial level (Fig. 5A). In addition, we examined the expression levels of *CiERF023* and *CiERF041* following the treatment with ACC, a precursor of ETH synthesis. Both *CiERF023* and *CiERF041* were quickly induced by ACC application and reached their highest levels at 48 h (Fig. 5B and C). Subcellular localization analyses revealed that both CiERF023 and CiERF041 are nuclear proteins, consistent with their functions as TFs (Fig. 5D and E).

The transient LUC reporter system was used to assess the transcriptional activation activities of the two TFs. For this purpose, the effectors of BD-CiERF023 and BD-CiERF041, along with a negative control (BD) and a positive control (BD-VP16), were co-infiltrated with the reporter vector containing GAL4-TATA into *N. benthamiana* leaves. Compared with the negative control, co-expression of BD-CiERF041 and the reporter resulted in noticeable elevation of the LUC intensity, which was otherwise profoundly decreased when BD-CiERF023 and the reporter were expressed together (Fig. 5F and G). Consistent with the fluorescence imaging, the ratio of LUC/REN was increased by infiltration of the reporter with BD-CiERF041, but significantly repressed by BD-CiERF023 (Fig. 5H and I). These results add further evidence to support that CiERF023 and CiERF041 act as a transcriptional repressor and an activator, respectively.

### *CiERF041* and *CiERF023* play opposite role in regulation of cold tolerance

**Figure 6:**
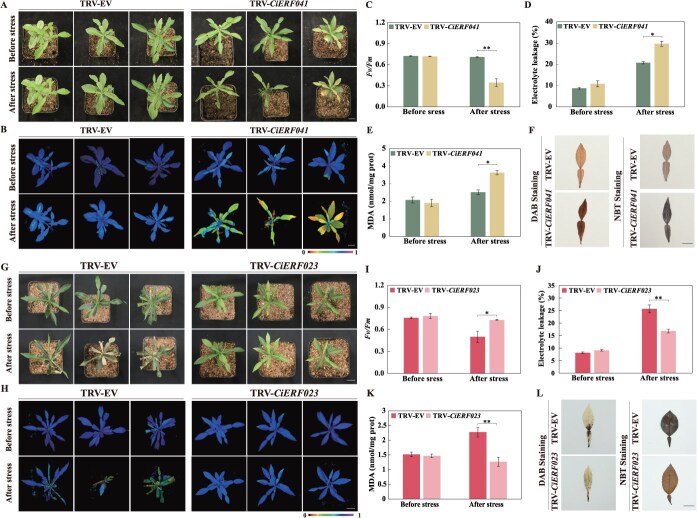
Knockdown of the *CiERF041* and *CiERF023* in Ichang papeda via VIGS technology influence the cold tolerance. (A–F) Silencing of *CiERF041* reduces its cold tolerance, (A and B) phenotypic and chlorophyll fluorescence images of TRV-*CiERF041* and TRV-EV control before and after low-temperature treatment, (C) *Fv/Fm* ratio, (D) EL, (E) MDA content, and (F) *in situ* visualization of H_2_O_2_ and O_2_^·−^ as revealed by DAB and NBT histochemical staining in leaves. (G–L) Knockdown of the *CiERF023* improves its cold tolerance, (G and H) phenotypic and chlorophyll fluorescence images of TRV-*CiERF023* and TRV-EV control before and after low-temperature treatment, (I) *Fv/Fm* ratio, (J) EL, (K) MDA content, (L) *in situ* visualization of H_2_O_2_ and O_2_^·−^ as revealed by DAB and NBT histochemical staining in leaves. Data are presented as mean ± SE (*n* = 3). Scale bars represent 1 cm for the images in the same panel.

In order to further investigate the biological roles of *CiERF041* and *CiERF023* in cold tolerance, they were separately silenced by using the VIGS method. In the VIGS plants, the expression levels of *CiERF041* and *CiERF023* were significantly repressed (Fig. S3). Under normal conditions, TRV-EV control and TRV-*CiERF041* plants grew well, with no significant differences in plant phenotype. However, after exposure to cold treatment and recovery, the TRV-*CiERF041* plants exhibited a stronger low-temperature sensitivity phenotype, as evidenced by noticeable leaf wilting and curling, significant impairment of chlorophyll fluorescence intensity and reduced *Fv/Fm* ratio, compared to the TRV-EV control (Fig. 6A–C). Additionally, the TRV-*CiERF041* plants exhibited significantly higher levels of EL and MDA, and accumulated prominently more ROS, including H_2_O_2_ and O_2_^·−^, in comparison with the TRV-EV control when subjected to the cold stress (Fig. 6D–F). Moreover, the expression levels of *CiHSP26.5* in the TRV-*CiERF041* plants were significantly lowered than those in the TRV-EV control (Fig. S4). In conclusion, silencing of *CiERF041* led to enhanced cold sensitivity and repression of *CiHSP26.5*, implying that it acts as a positive regulator of cold tolerance.

In addition, silencing of *CiERF023* was achieved via VIGS to generate TRV-*CiERF023* plants. Surprisingly, upon exposure to cold treatment and recovery, the TRV-*CiERF023* plants exhibited less serious cold damage and lower sensitivity than the TRV-EV control. Mild water-soaked wilting was observed on the apical young leaves of TRV-*CiERF023* plants, whereas serious leaf curling or even necrosis was present in the TRV-EV plants (Fig. 6G). Congruent with the plant phenotype, better chlorophyll fluorescence intensity and significantly higher *Fv/Fm* ratio, accompanied by lower levels of EL and MDA, were scored in the TRV-*CiERF023* plants when compared with the TRV-EV control (Fig. 6H–K). Additionally, lessened accumulation of ROS was noticed in the leaves of the TRV-*CiERF023* plants relative to the TRV-EV control (Fig. 6L). Furthermore, the expression levels of *CiHSP26.5* in the TRV-*CiERF023* plants were significantly higher than those in the TRV-EV control (Fig. S5). Collectively, silencing of *CiERF023* increased the expression of *CiHSP26.5* and rendered the plants more tolerant to cold stress, suggesting that *CiERF023* functions as a negative regulator of cold tolerance.

**Figure 7:**
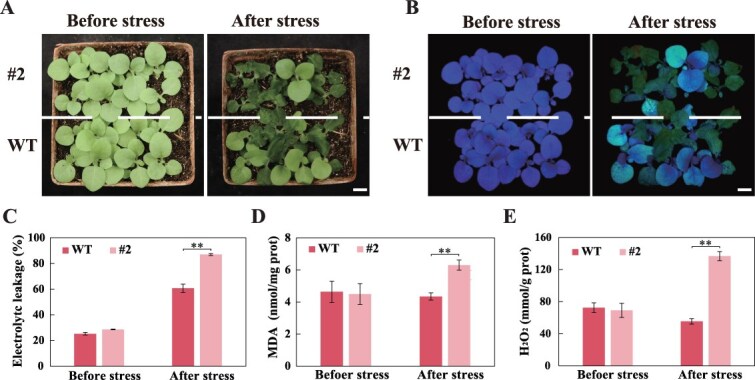
Overexpression of *CiERF023* suppresses the cold tolerance in transgenic tobacco. (A–E) Evaluation of cold tolerance in overexpressing tobacco, (A and B) phenotypic and chlorophyll fluorescence images, (C) EL, (D) MDA content, and (E) H_2_O_2_ content of the transgenic tobacco and WT before and after low-temperature treatment. Data are presented as mean ± SE (*n* = 3). Scale bars represent 1 cm for the images in the same panel.

In order to further validate the biological role of *CiERF023* in modulation of cold tolerance, *CiERF023* was ectopically expressed in tobacco to generate transgenic OE plants (Fig. S6). The WT and OE tobacco plants were morphologically similar when they were grown under normal conditions. However, when subjected to cold treatment, the majority of transgenic OE plants died, whereas the WT plants exhibited better performance (Fig. 7A). Consistently, weakened chlorophyll fluorescence intensity, concurrent with significantly higher levels of EL, MDA, and H_2_O_2_, was observed in the tobacco OE line compared to the WT (Fig. 7B–E). Overall, these findings indicate that ectopic overexpression of *CiERF023* in tobacco led to enhanced cold sensitivity, further supporting that *CiERF023* negatively regulated cold tolerance.

### CiERF023 represses *CiERF041* expression 

**Figure 8:**
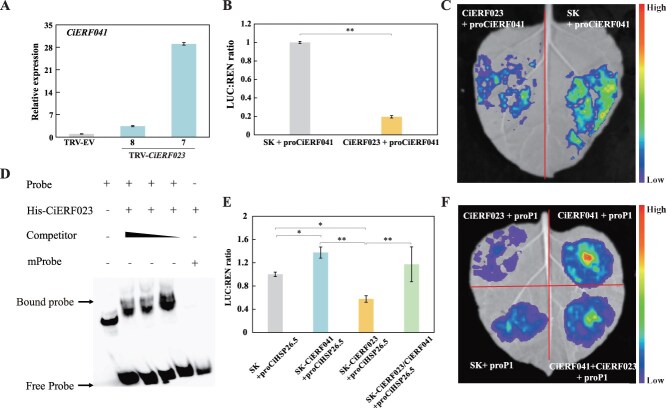
CiERF023 binds to the promoter of *CiERF041* and inhibits its expression, and analysis of their regulation pattern of CiHSP26.5. (A) The expression of *CiERF041* in TRV-EV control and TRV*-CiERF023* plants. (B and C) Dual LUC assay (B) and LUC bioluminescence imaging (C) were performed in tobacco, co-transformed with different effectors, including SK, SK-CiERF023, and the promoter of *CiERF041.* (D) Detection of CiERF023 binding to the promoter of *CiERF041* via EMSA. (E and F) Dual LUC assay (E) and LUC bioluminescence imaging (F) in tobacco co-transformed with the two effectors, SK-CiERF023 and SK-CiERF041, alone or together, and the promoter fragment of P1. Data are presented as mean ± SD (*n* = 3).

Under cold treatment, *CiERF023* and *CiERF041* showed distinct time-dependent expression patterns. *CiERF023* was upregulated at 12 h, maintained the elevated level at 24 h, and then downregulated at 72 h. In contrast, *CiERF041* remained unchanged at 12 and 24 h, and was only upregulated at 72 h (Fig. S7). Given the phase-specific differences in their expression patterns, we hypothesized a potential regulatory relationship between *CiERF023* and *CiERF041*. To verify this hypothesis, we analyzed *CiERF041* expression levels in TRV-*CiERF023* plants, in which *CiERF023* was prominently knocked down. The results indicate that the transcript levels of *CiERF041* in TRV-*CiERF023* plants were significantly higher than those in the TRV-EV control (Fig. 8A). To further confirm this assumption, we obtained a 2114-bp promoter sequence of *CiERF041* and identified a potential DRE sequence (ACCGAC). The transient LUC assay showed that co-expression of SK-CiERF023 and the reporter driven by the 2114-bp promoter sequence significantly decreased the promoter activity, as revealed by the reduction of the LUC/REN ratio relative to the control (Fig. 8B) and the weaker LUC fluorescence intensity (Fig. 8C). EMSA indicated that incubation of His-CiERF023 with the 39-bp probe synthesized based on the promoter sequence harboring the ACCGAC motif resulted in the formation of a protein-DNA complex, while inclusion of the competitors impaired the migration in a concentration-dependent manner. In addition, when the DRE sequence was mutated from ACCGAC to GAAAGA, the band shift was completely disrupted (Fig. 8D). These results demonstrate that CiERF023 is a transcriptional repressor of *CiERF041*. In addition, co-expression of SK-CiERF023 and SK-CERF041 with the reporter containing the P1 fragment of *CiHSP26.5* promoter slightly decreased the *CiHSP26.5* promoter activity when compared with the infiltration of SK-CiERF041 alone, but significantly increased relative to infiltration of SK-CiERF023 alone (Fig. 8E and F). These results indicate that the two ERFs may antagonize with each other to regulate CiHSP26.5.

## Discussion

In the face of recurrent extreme weather events, particularly low-temperature stress, plants have evolved a sophisticated and effective defense network to regulate their adaptability. On the one hand, by scavenging ROS [39, 40], degrading starch [41], and non-essential proteins [42], it provides raw materials and energy for the synthesis of defense compounds. On the other hand, they synthesize osmotic regulators, such as raffinose [27, 43], trehalose [44, 45], and proline [14], while increasing protective proteins [46–48] to safeguard structural and functional regulation. HSPs are a group of important protective proteins that are highly conserved in the evolutionary process of molecular chaperones. The network of HSPs has traditionally been associated with the heat stress response, but in fact it is a core component of multiple stress responses [49], and plays an irreplaceable role in the adaptation of animals and plants. Stress impedes normal cellular functions executed by proteins directly or indirectly, leading to protein denaturation and disruption of protein homeostasis. HSPs and other molecular chaperones participate in a variety of normal cellular processes, which include protein folding, assembly, transport, and degradation. The function of these proteins is to stabilize proteins and membrane structures, assist in protein refolding under stress conditions, and maintain cellular homeostasis by restoring normal protein conformations [20]. Collectively, they play a crucial role in protecting plants against stress.

HSPs play a crucial role in plant cold adaptation. Studies indicate that HSPs not only maintain protein homeostasis but also actively participate in transcriptional regulation, forming multi-level regulatory networks through epigenetic modifications and interactions with TFs. In terms of epigenetics, HSPs can regulate the stability of transcriptional products via RNA-level epigenetic modifications, establishing unique cold adaptation mechanisms. Under low-temperature stress, HSP70 is induced to preferentially bind m^6^A-modified RNA in a sequence-specific manner, forming granule-like condensates that stabilize the secondary structure of stress-related mRNAs and facilitate efficient translation of downstream genes [50]. In the cold-regulated transcriptional network, HSPs are regulated by multiple TFs through direct or indirect mechanisms. MaHsf24 acts as a negative regulator to suppress the expression of *HSPs* and reduces the fruit’s tolerance to low-temperature stress [16], and low temperatures induce nuclear translocation of NON-EXPRESSER OF PATHOGENESIS-RELATED GENES 1, where it interacts with heat shock TF A1 to activate HSPs expression [51]. These findings demonstrate the crucial role of HSPs in plant responses to cold stress, integrating into cold-sensing networks through multiple mechanisms. Although HSPs have been relatively well studied, the biological function and relevant cellular protective mechanism of *HSPs* under stress remain largely unknown [52]. The chloroplast-localized chaperone HSP21, to which *CiHSP26.5* is most closely related, protects the PSII complex through direct interaction with its core subunits, including D1 and D2 [21], and plays an essential role in maintaining the integrity of the thylakoid membrane system under heat stress [53]. Given that CiHSP26.5 shares extremely high sequence conservation with CiHSP21 and both of them are localized in chloroplasts, they are proposed to exhibit identical or similar biological functions. Notably, multiple sHSPs have been detected in citrus, and they may function together to exhibit better protection against abiotic stresses. However, there is also a possibility that some sHSPs exhibit functional redundancy in response to stressful conditions, which is a common regulatory strategy in plant stress responses [3]. Based on the RNA-seq data described earlier [37], the expression levels of five members in the HSP20 family, including *CiHSP26.5*, were significantly upregulated by cold stress (fold change ≥3) (Table S3). Meanwhile, prediction using the protein localization tool DeepLoc-2.0 [54] revealed that only CiHSP26.5 was predicted to be plastid-localized, which was further confirmed by subcellular localization assay. These results suggest that CiHSP26.5 may play a protective role in chloroplasts under cold stress.

ETH, as an important hormone in plant responses to abiotic stress, has been confirmed by numerous studies to participate in plant stress responses through the classical ETR–CTR1–EIN2–EIN3/ERF signaling pathway [55–57]. In cold responses, ETH exhibits species-specific functions and possesses bidirectional regulatory properties. In tomatoes [58] and *Arabidopsis* [59], exogenous application of ACC (1-aminocyclopropane-1-carboxylic acid) and ETH reduced cold tolerance. However, in trifoliate orange, *PtrERF9* is induced by ACC, the application of ACC results in an enhancement of the cold tolerance [5]. Despite the fact that research on the ETH signaling pathway is now relatively well-defined, studies on downstream genes or directly acted-upon substances within this pathway remain incomplete. In this study, it was similarly found that ACC treatment increased the mRNA abundance of *CiERF023* and *CiERF041*, suggesting that both TFs may regulate citrus cold tolerance by actively participating in the ETH signaling pathway.

ERFs, core members of the plant-specific AP2/ERF superfamily, function as a pivotal molecular basis for plant adaptation to environmental stresses and the regulation of developmental processes. It has been shown that the multifaceted regulatory functions and adaptable mechanisms of this system enable it to act in both an activator role, initiating target gene expression [60–62], and an inhibitor role, blocking transcription [31, 63]. These modes encompass both independent and cooperative regulation, forming a multilevel transcriptional regulatory network. Notably, individual ERFs can simultaneously perform dual regulatory functions. In tomatoes, SlERF.H5 and SlERF.H7 activate the expression of *SlCESA3*, maintaining cell wall integrity during fruit development, while simultaneously suppressing the expression of *GA20ox1* [64]. This dual functionality may enable internal trade-off of resource allocation between fruit development and plant growth. In *Citrus*, CiERF023 has been previously demonstrated to bind to the promoter of *CiACS4* and activate its expression [65]. Interestingly, this study confirms that CiERF023 also binds to the promoters of *CiERF041* and *CiHSP26.5*, leading to their transcriptional repression. These findings indicate that CiERF023 possesses dual transcriptional regulatory functions, acting as either an activator or a repressor depending on the specific context of its target genes. It is highly likely that CiERF023 plays a pivotal role in integrating multiple physiological pathways. Furthermore, we also observed a disparity in temporal expression pattern between *CiERF023* and *CiERF041*, as *CiERF023* was induced at early stage of cold treatment, while *CiERF041* was upregulated at later stage. Two possibilities may explain such difference, although others cannot be fully ruled out. First, *CiERF023* and *CiERF041* may be regulated by two different upstream regulators that exhibited opposite regulation dynamics under cold stress. Second, *CiERF023* and *CiERF041* were regulated by an unknown common player that acted as both a repressor and an activator of ERFs, depending on the cold stress duration. However, these speculations and the precise reasons warrant further exploration in the future.

**Figure 9:**
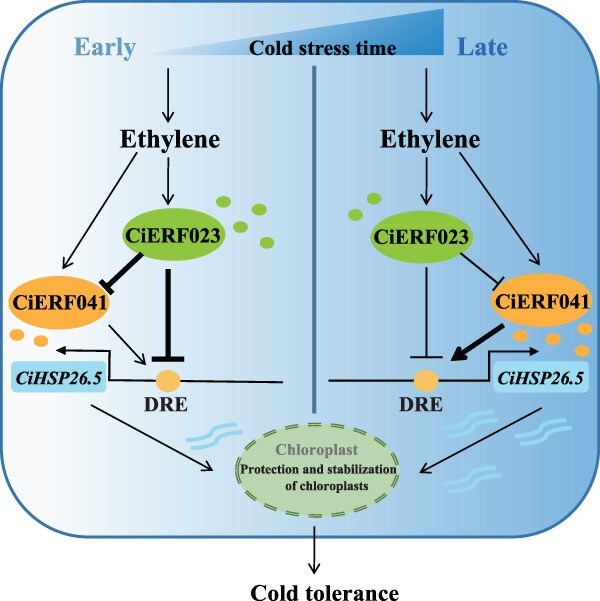
A proposed working model for regulation of *CiHSP26.5* by ERFs under cold stress in Ichang papeda. In the early stage of cold stress, CiERF023 serves as a transcription repressor, inhibiting the transcription of *CiERF041* and *CiHSP26.5.* With prolonged low-temperature stress, the expression of *CiERF023* decreases, relieving its repression on the two genes. In addition, CiERF041 acts as a transcription activator, triggering an active response to drive substantial expression of *CiHSP26.5*, thereby working to promote cold stress adaptation and tolerance.

A delicate balance between environmental stress responses and growth, development, and energy metabolism exists throughout the plant life cycle. This is particularly evident during cold stress, where the activation and maintenance of cold tolerance is an energetically costly process that diverts energy and resources away from growth and development [66, 67]. In the present study, we also identified a temporal and hierarchical balanced regulatory network underlying cold stress responses. During the early stage of cold stress, *CiERF023* acts as a pioneer responsive factor and functions as a ‘molecular brake’ to fine-tune the stress response. Specifically, *CiERF023* adopts a two-layered regulatory mechanism. On the one hand, it directly binds to the promoter of *CiHSP26.5* to repress its expression. On the other hand, it represses the transcription of *CiERF041*, thereby fundamentally attenuating the activating effect of CiERF041 on *CiHSP26.5*. This regulation effectively prevents oxidative stress and metabolic imbalance that would otherwise be induced by the premature and excessive activation of the CiERF041-*CiHSP26.5* regulatory module. During the late stage of cold stress, the expression level of *CiERF023* decreases, alleviating its inhibitory regulation on downstream targets. Concomitantly, the CiERF041-*CiHSP26.5* regulatory module is activated to exert stress-resistant protective effects (Fig. 9). This precisely orchestrated regulatory strategy may accomplish the dynamic balance between cellular energy metabolism and stress response processes.

## Materials and methods

### Plant materials and growth conditions

Ichang papeda was collected from the germplasm resource repository at Huazhong Agricultural University, and lemon was obtained from a production base in Anyue County of Sichuan, China. They were germinated in pots containing soil mix under conditions of 26°C, long-day photoperiod (16 h of light:8 h of darkness). For cold treatment, Ichang papeda plants grown at room temperature were transferred to a low-temperature (4°C) incubator. Leaves were harvested at specified time points, rapidly frozen in liquid nitrogen, and stored at −80°C for further analysis. For hormone treatment, plants were incubated in a solution containing 100 μM ACC and H_2_O.

### RNA extraction and RT-qPCR analysis

To investigate gene expression, total RNA was extracted using a commercial RNA extraction kit (RN33; Aidlab Biotech Co. Ltd, Beijing, China), followed by cDNA synthesis with HiScript III Reverse Transcriptase (R323, Vazyme, Nanjing, China) according to the manufacturer’s instructions. RT-qPCR was performed using ChamQ Universal SYBR qPCR Master Mix (Q711, Vazyme, Nanjing, China) on an ABI 7500 system (Applied Biosystems, Foster City, CA, USA). The amplification reaction was pre-denatured at 95°C for 5 minutes, followed by 40 cycles, each cycle including 10 seconds of denaturation at 95°C, 30 seconds of annealing at 60°C. The reaction system was 10 μl, containing 5 μl SYBR Green PCR Master Mix, 0.2 μl of primers, and 200 ng of cDNA. Each sample was replicated three times. Actin was used as the internal reference gene in all experiments to standardize the expression levels of the measured genes according to the 2^–ΔΔCT^ algorithm [68]. The primer sequences used in this study are listed in Table S1.

### Subcellular localization and sequence analysis

The coding sequences (CDS) of *CiHSP26.5*, *CiERF023*, and *CiERF041* without the stop codons were inserted into the 101LYFP vector harboring a YFP, driven by CaMV 35S promoter. The recombinant plasmid or EV was co-transformed with the nuclear marker VirD2NLS mCherry into tobacco (*N. benthamiana*) leaves through *Agrobacterium*-mediated transformation. Two days later, the YFP fluorescence signal was observed using a laser scanning confocal microscope (Leica TCS-SP8, Wetzlar, Germany). In addition, phylogenetic tree construction of *CiHSP26.5* was performed using the software MEGA11 (Molecular Evolutionary Genetics Analysis version 11) [69].

### Vector construction and plant transformation

For generation of *CiHSP26.5*-overexpression lines, the full-length CDS without stop codon was recombined into the pGWB411 vector (with a Flag tag driven by the CaMV 35S promoter) through the gateway BP (11789100, Thermo Fisher, CA, USA) and LR (11791020, Thermo Fisher, CA, USA) reactions. For generation of *CiERF023-*overexpression line, the full-length CDS without stop codon was recombined into the pBI121 vector. Subsequently, the transformation of lemon and tobacco was achieved following earlier report [70].

For VIGS, partial CDS fragments of *CiHSP26.5* (101–357 bp, 5′-CDS regions)*, CiERF023* (284–525 bp, 5′-CDS regions) and *CiERF041* (324–582 bp, 5′-CDS regions) were amplified and integrated into the tobacco rattle virus-based vector 2 plasmid (TRV2). The TRV2, TRV1, and the fusion constructs (TRV-*CiHSP26.5*, TRV-*CiERF023*, and TRV-*CiERF041*) were transformed into *GV3101*, respectively. In the light of the earlier protocols [71, 72], the bacterial suspension of TRV1 was mixed with TRV2 or TRV-*CiHSP26.5*/TRV-*CiERF023*/TRV-*CiERF041* in a 1:1 ratio, and transformed into 1-month-old Ichang papeda etiolated seedlings. The infected seedlings were cultivated in the dark for 3 days, then transplanted to pots with soil. After 1 month of growth, the positive plants were selected via genomic PCR and RT-qPCR assays for further detection.

### Cold tolerance assay

For the cold treatment of transgenic tobacco, 1-month-old transgenic tobacco and WT seedlings exposed to a temperature of −4°C until the onset of cold damage phenotypes was observed, followed by growth recovery for 24 h at room temperature. In another experiment, the transgenic lemon OE lines and WT with nearly consistent growth status were treated at −4°C treatment until distinct freeze damage phenotypes were observed, and then shifted to ambient temperature for 24 h. Furthermore, the VIGS plants of TRV-*CiHSP26.5*, TRV-*CiERF023*, TRV-*CiERF041*, and their TRV-EV control were maintained at −4°C until distinct cold-damage phenotypes became apparent, followed by growth recovery at ambient temperature for 24 h. Plant phenotype and chlorophyll fluorescence imaging, captured using a chlorophyll fluorimeter (IMAGING-PAM, Walz, Germany), were scored before and after the cold treatment, and leaves were sampled after freezing treatment for further analysis.

### Physiological measurements

EL was measured by investigating relative conductance as previously described [73]. The ratios of *Fv/Fm* were calculated based on Imaging WinGegE software. The MDA content and H_2_O_2_ content were measured by commercial kits (A003 for MDA, A064 for H_2_O_2_, A405 for protein, Nanjing Jiancheng Bioengineering Institute, Nanjing, China) following the instructions. In addition, the leaf samples were stained with DAB and NBT to detect the *in situ* accumulation of H_2_O_2_ and O_2_^·−^, respectively, following the previous method [74].

### Yeast one-hybrid screening of complementary DNA library

The P1 fragment of *CiHSP26.5* (−1 to −1069 bp) was cloned into the pAbAi vector, and the resulting construct was linearized with the restriction enzyme *Bst*BI and integrated into the Y1H Gold yeast strain via homologous recombination to generate the bait. Screening of a cDNA library was performed with the Matchmaker Gold Y1H Library Screening System (Cat. No. 630491; Takara, Kyoto, Japan) with stringent selection with 40 ng/ml AbA (AbA; Coolaber, Beijing, China). The resulting putative clones were sequenced and subjected to bioinformatics annotations.

For Y1H assay, the CDS of *CiERF023* and *CiERF041* were amplified and fused to the pGADT7 vectors to generate prey vectors. Then the prey vectors were transformed into the bait using the Matchmaker Y1H library Screening System (Clontech) following the manufacturer’s protocol. The yeast cells transformed with the prey and bait vectors, the positive (pGADT7-p53 + p53-AbAi) or the negative (pGADT7 + P1-AbAi) controls were diluted and streaked on SD/-Ura/-Leu medium added with or without 150 ng/ml AbA. After a 3-day growth at 30°C, the yeast cell growth was recorded.

### Transient dual luciferase assay

The transcriptional activation activity was examined by imaging firefly LUC fluorescence *in vivo*. To this end, the effector plasmids were constructed by fusing the CDS of *CiERF023* and *CiERF041* to GAL4 DBD vector. A reporter plasmid comprising an LUC reporter gene under the regulation of a TATA-box and 5 × GAL4 elements was co-infiltrated into tobacco leaf with CiERF023-GAL4 DBD or CiERF041-GAL4 DBD vector. VP16 and a non-modified vector were used as the positive and negative control, respectively.

For interaction verification analysis, the full-length CDS of *CiERF023* and *CiERF041* were insert into pGreenII 62-SK to generate SK-CiERF023 and SK-CiERF041 effectors, while the P1 fragment of *CiHSP26.5* promoter was cloned into the pGreenII 0800-LUC vector to generate a reporter. The vectors were transfected into *Agrobacterium tumefaciens tumefaciens* GV3101, which was then used to infiltrate *N. benthamiana* leaves. After 2-day incubation under darkness, LUC activity was measured by analyzing leaf samples using the Dual-Luciferase Reporter Assay Kit (E1910, Promega, WI, USA) following the manufacturer’s protocol. In addition, 1 mM D-luciferin potassium salt (Coolaber CL6930, Beijing, China) was applied on the underside of the leaves before the observation of the LUC fluorescence using the Night Shade LB985 chemiluminescent imaging system (Berthold, Germany) controlled by Indigo software.

### Electrophoretic mobility shift assay

The full-length CDS of *CiERF023* and *CiERF041* were cloned into the pHMGWA vector containing a His tag, and the vectors were transformed into *Escherichia coli* Rosetta (DE3) competent cells (Weidi Biotechnology, China). The recombinant proteins were induced at 16°C for 18 h with 0.5 mM isopropyl-β-D-1-thiogalactopyranoside, and purified in its native form using Ni-NTA agarose (Qiagen, Hilden, Germany) according to the manufacturer’s instructions. The probe containing original *CiHSP26.5* promoter motifs DRE element (GCCGAC) and mutated DRE elements (AAAAAA), and another probe containing original *CiERF041* promoter motifs DRE element (ACCGAC) and mutated elements (GAAAGA) were synthesized and labeled with biotin (TianYi HuaYu Gene Technology, Wuhan, China). Unlabeled probe of same genuine sequence was used as the competitor. The probes were incubated with the fusion protein in a 10 μl reaction solution. EMSA was carried out using a Chemiluminescent EMSA Kit (GS009, Beyotime Biotechnology, Shanghai, China) according to the manufacturer’s protocols. Visualization was conducted using the Chemiluminescence Nucleic Acid Detection Module (Thermo Fisher Scientific). Probes are shown in Table S1.

### Statistical analysis

The data of RT-qPCR and LUC/REN ratio are shown as means ± SD, and the data of stress-related physiological measurements are shown as means ± SE. They were processed using SPSS software (SPSS Statistics 17.0; SPSS Inc., Chicago, IL, USA). Analysis of variance (ANOVA) was used to analyze the statistical difference followed by the LSD test (^*^P < 0.05; ^**^P < 0.01).

## Supplementary Material

Web_Material_uhag131

## Data Availability

The data supporting this article is accessible within the text, as well as in the supplementary resources provided online.
