# Supplementary material for: The transcriptional cascade CiERF023-CiERF041 of Citrus ichangensis regulates small heat shock protein gene CiHSP26.5 to modulate cold tolerance
Source: Hortic Res. 2026 Apr 7;13(8):uhag131. doi: 10.1093/hr/uhag131 (PMC13437076; doi:10.1093/hr/uhag131)
Supplement: Supplementary file 1 [file web_material_uhag131.zip › Supplementary figures0301.doc]

**The CiERF023*-*CiERF041 cascade regulates *CiHSP26.5* to modulate cold tolerance in *Citrus***

**Xi Zeng^1^, Lele Chu^1^, Wenqiang Hu^1^, Yilei Wang^1^, Yike Zeng^1^, Peng Xiao^1^, Jing Qu^1^,** [**Bachar Dahr**](https://onlinelibrary.wiley.com/authored-by/Dahro/Bachar)**o^1^, Wei Li^1^, Shiping Zhu^3^, Chunlong Li^1,2^, Ji-Hong Liu^1,2,*^**

**Figure legends**

Figure S1. RT-qPCR identification of the TRV-*CiHSP26.5* plants.

Figure S2. RT-qPCR identification of transgenic lemon (*C. limon*) plants and tobacco (*N. tabacum*) plants overexpressing *CiHSP26.5*.

Figure S3. RT-qPCR identification of the TRV-*CiERF041* plants and TRV-*CiERF023* plants.

Figure S4. RT-qPCR identification of the expression of *CiHSP26.5* in TRV-EV control and TRV-*CiERF041* plants.

Figure S5. RT-qPCR identification of the expression of *CiHSP26.5* in TRV-EV control and TRV*-CiERF023* plants.

Figure S6. RT-qPCR identification of tobacco (*N. tabacum*) overexpressing *CiERF023* line.

Figure S7. The Fragments Per Kilobase (FPKM) values of *CiHSP26.5*, *CiERF023* and *CiERF041* under cold treatment.

**Figure S1. RT-qPCR identification of the TRV-*CiHSP26.5* plants.** Transcript abundance of TRV-*CiHSP26.5* lines relative to the TRV empty vector control. Error bars represent means ± standard deviation (n = 3).

**Figure S2. RT-qPCR identification of transgenic** **lemon** **(*C. limon*) plants and tobacco** **(*N. tabacum*) plants overexpressing *CiHSP26.5*.** Analysis of *CiHSP26.5* expression in lemon (A) and tobacco (B) wild type (WT) and overexpressing lines by RT-qPCR. Error bars represent means + standard deviation (n = 3).

**Figure S3. RT-qPCR identification of the TRV-*CiERF041* plants and TRV-*CiERF023* plants.** Transcript abundance of TRV-*CiERF041* plants (A) and TRV-*CiERF023* plants (B) relative to the TRV empty vector control. Error bars represent means ± standard deviation (n = 3).

**Figure S4. RT-qPCR identification of the expression of *CiHSP26.5* in TRV-EV control and TRV-*CiERF041* plants.** Analysis of *CiHSP26.5* relative expression in TRV-EV and TRV-*CiERF041* plants by RT-qPCR. Error bars represent means ± standard deviation (n = 3).

**Figure S5. RT-qPCR identification of the expression of *CiHSP26.5* in TRV-EV control and TRV*-CiERF023* plants*.*** Analysis of *CiHSP26.5* relative expression in TRV-EV and TRV-*CiERF023* by RT-qPCR. Error bars represent means ± standard deviation (n = 3).

**Figure S6. RT-qPCR identification of tobacco (*N. tabacum*) overexpressing *CiERF023* line.** Analysis of *CiERF023* expression in to tobacco WT and OE line by RT-qPCR. Error bars represent means + standard deviation (n = 3).

**Figure S7. The Fragments Per Kilobase (FPKM) values of *CiHSP26.5*, *CiERF023* and *CiERF041* under cold treatment.** Changes in FPKM values for *CiERF041*, *CiERF023*, and *CiHSP26.5* at different time points during low-temperature treatment. Error bars represent means ± standard deviation (n = 3).
