# Supplementary material for: The transcriptional cascade CiERF023-CiERF041 of Citrus ichangensis regulates small heat shock protein gene CiHSP26.5 to modulate cold tolerance
Source: Hortic Res. 2026 Apr 7;13(8):uhag131. doi: 10.1093/hr/uhag131 (PMC13437076; doi:10.1093/hr/uhag131)
Supplement: Supplementary file 1 [file web_material_uhag131.zip › Supplementary Tables.doc]

**The CiERF023*-*CiERF041 cascade regulates *CiHSP26.5* to modulate cold tolerance in *Citrus***

**Xi Zeng^1^, Lele Chu^1^, Wenqiang Hu^1^, Yilei Wang^1^, Yike Zeng^1^, Peng Xiao^1^, Jing Qu^1^,** [**Bachar Dahr**](https://onlinelibrary.wiley.com/authored-by/Dahro/Bachar)**o^1^, Wei Li^1^, Shiping Zhu^3^, Chunlong Li^1,2^, Ji-Hong Liu^1,2,*^**

Table S1. List of primers used in the study.

Table S2. List of the genes identified from Y1H library screening.

Table S3. FPKM values and localization of cold-induced HSP20 family members.

| **Table S1. List of primers used in the study.** | |
| --- | --- |
| qPCR | |
| RT-qPCR-*CiHSP26.5*-F | CCGGTAACCAAGGAACCCAA |
| RT-qPCR-*CiHSP26.5*-R | CCCCGGAAAAGCCATCGTAT |
| RT-qPCR-*CiERF041*-F | CCGAGTTTGGGGTCGAGTTA |
| RT-qPCR-*CiERF041*-R | CCAAGTCCAAGTCCAAGTCCA |
| RT-qPCR-*CiERF023*-F | GCACATGCCGGACTCATTTC |
| RT-qPCR-*CiERF023*-R | TTCCTGTGGCTGTGCATCTT |
| Gene expression and cloning | |
| FL-*CiHSP26.5*-F | ATGGCTGCAACATTATCTATCTCAT |
| FL-*CiHSP26.5*-R | CAGAATCTGAACATCAATGACCTTG |
| FL-*CiERF041*-F | ATGAGTGAACCGCAAAACTCATCGGAA |
| FL-*CiERF041*-R | GGAATCCCACAACAAACCTTCTCCAA |
| FL-*CiERF023*-F | ATGGAGCAACAACCATACACATACACTGAACATG |
| FL-*CiERF023*-R | CAAACATGCCATGGACGGCGGCTGCTGTTCCT |
| YFP101-*CiHSP26.5*-F | ATGGGATCTACTAGTGAATTCATGGCTGCAACATTATCTATCTCAT |
| YFP101-*CiHSP26.5*-R | GGGGGTACCGTCGACGGATCCCAGAATCTGAACATCAATGACCTTG |
| YFP101-*CiERF041*-F | ATGGGATCTACTAGTGAATTCATGAGTGAACCGCAAAACT |
| YFP101-*CiERF041*-R | GGGGGTACCGTCGACGGATCCGGAATCCCACAACAAACCT |
| YFP101-*CiERF023*-F | ATGGGATCTACTAGTGAATTCATGGAGCAACAACCATACACATACACTG |
| YFP101-*CiERF023*-R | GGGGGTACCGTCGACGGATCCCAAACATGCCATGGACGGCGGCT |
| TRV2-*CiHSP26.5*-F | AGAAGGCCTCCATGGGGATCCTACCGGCAACGTCAAGGAAA |
| TRV2-*CiHSP26.5*-R | TGTCTTCGGGACATGCCCGGGCCCCGGAAAAGCCATCGTAT |
| TRV2--*CiERF041*-F | AGAAGGCCTCCATGGGGATCCTCCTAACTCTCTCGCCCCACGT |
| TRV2--*CiERF041*-R | TGTCTTCGGGACATGCCCGGGAAACCAAGGCGGCGTGTACATCCAA |
| TRV2--*CiERF023*-F | AGAAGGCCTCCATGGGGATCCTTCCTGACAAGGTTGAGGCCTTGCC |
| TRV2--*CiERF023*-R | TGTCTTCGGGACATGCCCGGGTTCATCAATCCACGTGGACGTGGTAT |
| pDONR221-CiERF023-F | GGGGACAAGTTTGTACAAAAAAGCAGGCTCCATGGAGCAACAACCATACACATACACTGAACATG |
| pDONR222-CiERF023-R | GGGGACCACTTTGTACAAGAAAGCTGGGTCCAAACATGCCATGGACGGCGGCTGCTGTTCCT |
| pDONR221-CiERF041-F | GGGGACAAGTTTGTACAAAAAAGCAGGCTCCATGAGTGAACCGCAAAACTCATCGG |
| pDONR222-CiERF041-R | GGGGACCACTTTGTACAAGAAAGCTGGGTCGGAATCCCACAACAAACCTTCTCCA |
| Y1H assay | |
| pABAi-Pro*CiHSP26.5*-F | CTTGAATTCGAGCTCGGTACCATGGACGGGTAGGTATTTTGCCGAC |
| pABAi-Pro*CiHSP26.5*-R | ATACAGAGCACATGCCTCGAGGCACTTGAGAGAATCGATCGATG |
| pGADT7-CiERF023-F | GATTACGCTCATATGATGGAGCAACAACCATACACATACACTG |
| pGADT7-CiERF023-R | ACCCGGGTGGAATTCCAAACATGCCATGGACGGCGGCT |
| pGADT7-CiERF041-F | GATTACGCTCATATGATGAGTGAACCGCAA |
| pGADT7-CiERF041-R | ACCCGGGTGGAATTCGGAATCCCACAACA |
| EMSA | |
| *CiHSP26.5*-P1- probe | ACGGGTAGGTATTTTGCCGACCAAATGCCAAGTAGT |
| *CiHSP26.5-*mP1-probe | ACGGGTAGGTATTTTAAAAAACAAATGCCAAGTAGT |
| *CiERF041*- probe | ATTACATAGAAATTAACCGACCATACATGAGGAAGGGGC |
| *CiERF041-m*-probe | ATTACATAGAAATTAGAAAGACATACATGAGGAAGGGGC |
| LUC assay | |
| 0800-Pro*CiHSP26.5*-FL-F | GTCGACGGTATCGATAAGCTTATGGACGGGTAGGTATTTTGCCGAC |
| 0800-Pro*CiHSP26.5*-FL-R | CGCTCTAGAACTAGTGGATCCGCACTTGAGAGAATCGATCGATG |
| 0800-Pro*CiERF04*-FL-F | GTCGACGGTATCGATAAGCTTAATAAATATACGTGCGTACA |
| 0800-Pro*CiERF041*-FL-R | CGCTCTAGAACTAGTGGATCCCATCTTGTAGTCGTTGTTGATGT |
| 62 SK-*CiERF023*-F | CGCTCTAGAACTAGTGGATCCATGGAGCAACAACCATACACATACACTG |
| 62 SK-*CiERF023*-R | GATAAGCTTGATATCGAATTCCAAACATGCCATGGACGGCGGCT |
| 62 SK-*CiERF041*-F | CGCTCTAGAACTAGTGGATCCATGAGTGAACCGCAAAACTCATCGGAA |
| 62 SK-*CiERF041*-R | GATAAGCTTGATATCGAATTCGGAATCCCACAACAAACCTTCTCCAA |

| **Table S2. List of the genes identified from Y1H library screening.** | |
| --- | --- |
| Gene ID | Gene Description |
| *Ci113400.1* | Ethylene-responsive transcription factor *ERF041.* |
| *CI022320.1* | Ethylene-responsive transcription factor *ERF023.* |
| *Ci002910.1* | Transcription factor PIF1-related. |
| *CI191070.2* | Auxin response factor |
| *Ci108610.1* | AN1-like Zinc finger |

| **Table S3. FPKM values and localization of cold-induced HSP20 family members.** | | | | | | | | | | | | | | |
| --- | --- | --- | --- | --- | --- | --- | --- | --- | --- | --- | --- | --- | --- | --- |
| ID | YCC-0H1 | YCC-0H2 | YCC-0H3 | YCC-12H1 | YCC-12H2 | YCC-12H3 | YCC-24H1 | YCC-24H2 | YCC-24H3 | YCC-72H1 | YCC-72H2 | YCC-72H3 | Subcellular localization prediction |  |
| *Ci112110* | 3.3 | 3.42 | 3.98 | 6.96 | 3.77 | 4.42 | 2.54 | 3.67 | 3.4 | 11.7 | 10.47 | 10.76 | Plastid |  |
| *Ci264590* | 2.11 | 2.2 | 2.4 | 5.83 | 6.85 | 6.35 | 10.26 | 10.33 | 7.15 | 3.95 | 4.36 | 5.34 | mitochondrion |  |
| *Ci277670* | 0.8 | 0.34 | 0.54 | 2.85 | 2.2 | 2.99 | 3.28 | 2.21 | 1.88 | 7.07 | 6.44 | 5.84 | mitochondrion |  |
| *Ci247260* | 9.41 | 9.44 | 8.01 | 10.48 | 10.85 | 10.56 | 9.61 | 14.08 | 12.5 | 91.96 | 85.45 | 88.52 | mitochondrion |  |
| *Ci246470* | 11.15 | 13.72 | 12.57 | 36.69 | 37.25 | 37.95 | 74.78 | 87.49 | 92.89 | 140.17 | 146.47 | 145.16 | cytoplasm |  |
